# *Scedosporium apiospermum* endopthalmitis treated early with intravitreous voriconazole results in recovery of vision

**DOI:** 10.1007/s12348-012-0063-0

**Published:** 2012-02-28

**Authors:** Monika Paroder Belenitsky, Catherine Liu, Irena Tsui

**Affiliations:** 1 Department of Ophthalmology, Montefiore Medical Center, Bronx, NY USA; 2 Jules Stein Eye Institute, Los Angeles, CA USA

**Keywords:** Endogenous endopthalmitis, *Scedosporium apiospermum*

## Abstract

**Aim:**

The purpose of this study is to report a case of endogenous endopthalmitis caused by *Scedosporium apiospermum* with a favorable outcome and review previously reported cases, their treatment regimens and outcomes.

**Methods:**

An 83-year-old man with diabetes mellitus, no other immunocompromising risk factors, and a history of *S*. *apiospermum* endopthalmitis in the left eye developed endopthalmitis in the right eye. Within 72 h of presentation, he was treated with a pars plana vitrectomy and intravitreal voriconozole.

**Results:**

Vitreous cultures confirmed *S*. *apiospermum*. The patient responded to treatment, with a favorable outcome and full recovery of vision.

**Conclusions:**

Recognition of *S*. *apiospermum* endopthalmitis and appropriate early intervention with pars plana vitrectomy and intravitreal voriconozole can lead to a favorable outcome with restoration of visual acuity.

## Introduction

*Scedosporium apiospermum* is an opportunistic fungus that can affect the eye, presenting as keratitis, chorioretinitis, or endophthalmitis, often with devastating consequences [1]. Disseminated life-threatening disease and endogenous endophthalmitis are generally seen in immunocompromised patients; however, immunocompetent individuals can also be affected with exogenous endophthalmitis from trauma.

This species of *Scedosporium*, the anamorph (asexual state) of *Pseudallescheria boydii*, is a ubiquitous filamentous fungus, found in soil, sewage, and polluted water [1]. The subclassification of this genetically heterogeneous species makes medical literature confusing. Synomorphs of *S*. *apiospermum* include *Monosporium apiospermum*, *Monosporium sclerotiale*, *Indiella americana*, *Acremoniella lutzi*, and *Polycytella hominis* [1]. In addition, the number of cases may be underreported or misdiagnosed because it is clinically indistinguishable from “the great imitator” and more commonly occurring fungus, *Asperigillus fumigatus* [2, 3].

Although uncommon, *S*. *apiospermum* endophthalmitis has been reported in the literature, with nine cases prior to 2005 reviewed by Larocco et al. [4–17], all with poor outcomes. We review additional reported cases of endogenous *S*. *apiospermum* endopthalmitis, including comorbidities, treatment modality, and final outcomes (Table 1). Furthermore, we report a unique case of our own that was detected early and treated aggressively, leading to a good outcome.

**Table 1:** Review of additional reported cases of endogenous endopthalmitis caused by *S. apiospermum* not reviewed by Larocco et al.

Author	Year	Gender	Age	Comorbidities	Laterality	Culture source	Treatment	Other sites of infection	Outcome
McKelvie et al. [9]	2001	F	38	AML, neutropenia	OU	Blood and vitreous	Intraocular AmpB, Foscarnet, Vanco (dx species postmortem)	Blood	CF
Figueroa et al. [13]	2004	M	44	Post-kidney transplant; on mycophenolate, tacrolimus, and corticosteroids	OS	Vitreous	Vitrectomy, PO Vori	None	CF
Larocco et al. [12]	2005	F	28	Presumed sinusitis, sepsis 2/2, pseudomemebranous colitis	OD	Vitreous	AmpB IVT and systemic, followed by itraconazole when dx of *S*. *apiospermum* was made	None	Evisceration
Musk et al. [4]	2006	M	57	Lung transplant due to alpha1-anti-trypsin deficiency, on cyclosporine, azothioprine, and prednisolone	OS	Vitreous and epididymis	PO, IVT, and topical Vori	Lung and skin nodules, epididymoorchitis	Poor vision, remains on voriconozole with no evidence of disease
		M	63	Lung transplant for interstitial pneumonitis on high-dose steroids	OS	PCR retinal biopsy, vitreous culture	Intravitreous Vori and AmpB; PO Vori	Kidney, urine	No recovery of vision, survived
Jain et al. [17]	2007	F	59	Pre-B cell acute LL, neutropenia	OS	Vitreous	Vori IVT and PO	Blood and lung	Enucleation, death from sepsis; NLP
		F	37	Pre-B cell acute LL	OS	Vitreous	IVT voriconozole, AmpB	Lung	Endopthalmitis stabilized; multiorgan system failure
		F	21	Wegener’s granulomatosis; corticosteroids and cyclophosphamide	OU	Vitreous	IVT AmpB, itraconazole	None	No visual recovery, CNS involvement
Chen et al. [7]	2007	M	56	Post-lung transplant; immunosuppresive Rx	OS	Vitreous	Vitrectomy, IV and IVT Vori	None	Enucleation; secondary scleritis; LP
		M	62	Post-lung transplant	OS	Vitreous	PO Vori, IV AmpB, PO terbinafine, IVT Vori	None	Enucleation; LP
Shankar et al. [5]	2007	M	61	DM, HTN	OD	Aqueous aspirate	Anterior chamber wash and IVT Vori	None	Resolution of vitreous exudates
Ikewaki et al. [11]	2009	M	58	DM; sub-Tenon’s triamcinolone injection	OS	Vitreous	Topical and IVT irrigation with Vori	None	Improvement to baselineImprovement to baseline (0.02 to 0.5)
Present case	2011	M	83	DM	OD	Vitreous	IV and IVT Vori	None	Stable at 20/40

*AmpB* amphotericin B, *Vori* voriconozole, *IVT* intravitreal, *DM* diabetes mellitus, *HTN* hypertension, *AML* acute myeloid leukemia, *PO* per oral, *dx* diagnosis

### Case report

An 83-year-old monocular male presented with complaints of new floaters in his right eye. His visual acuity was counting fingers, intraocular pressure was 8 mmHg with 3+ cells in his anterior chamber, and a dense vitritis. Ultrasound confirmed vitritis with an attached retina. The patient was seen by a referring ophthalmologist the day before with similar complaints of floaters with visual acuity of 20/40 due to nuclear sclerosis.

The patient had been no light perception in his left eye for over a year due to endogeneous endophthalmitis from a fungal lung lesion. At that time, both vitreous and lung biopsies grew out *S*. *apiospermum*. Other than diabetes, a thorough infectious disease work-up revealed no other immunocompromising risk factors. The lung lesions were unchanged after a course of treatment, and the patient’s family did not want further work-up such as a re-biopsy due to the patient’s age. The patient was prescribed a maintenance dose of voriconazole 200 mg PO QD, but he was noncompliant and stopped taking his medicine.

At the time of presentation to our clinic, the patient had symptoms in his right eye for less than 24 h. An intravitreal biopsy was performed in conjunction with intravitreal voriconazole (100 μg/0.1 ml). Vitreous biopsy at this time grew out *S*. *aspiospermum*. The patient was also started on intravenous voriconazole. After 48 h with no improvement, the patient was brought to the operating room for a 23-gauge pars plana vitrectomy and repeat intravitreal voriconazole (100 μg/0.1 ml) injection. There were no intraretinal lesions seen at the time of surgery, although the view was hazy. The patient did not receive any intravitreal or oral steroids. His inflammation subsided slowly over the course of 3 weeks with moxifloxacin and prednisolone acetate eye drops. One month after presentation, his visual acuity returned to 20/40.

## Discussion

*S*. *apiospermum* endophthalmitis presents with an aggressive clinical course, oftentimes requiring enucleation. Treatment is particularly challenging due to resistance to many antifungal agents. Here, we report a case of endogenous *S*. *apiospermum* endopthalmitis in a diabetic patient that responded favorably to voriconazole with full restoration of visual acuity. To our knowledge, this is the third reported case of successful treatment outcome for endogenous endophthalmitis due to this fungal species [5, 11]. Our case is unique in that voriconazole combined with early surgical intervention led to a favorable outcome.

Two cases of successful treatment outcomes for *S*. *apiospermum* endopthalmitis have previously been described. In 2007, Shankar et al. reported a case a case of endogenous endophthalmitis from an unknown source in a 61-year-old man with diabetes mellitus (DM). Anterior chamber biopsy was positive for *S*. *apiospermum* and the patient was treated with intravitreal amphotericin B and voriconazole with restoration of vision [5].

Two years later, another case of *S*. *apiospermum* endophthalmitis with a good outcome was published by Ikewaki et al. They described a 58-year-old man with DM who developed exogenous endopthalmitis following sub-Tenon’s injection with triamcinolone acetonide for treatment of macular edema. The patient was not diagnosed or treated until 5 months after the sub-Tenon’s injection, when a periocular abscess was drained with cultures revealing *S*. *apiospermum*. By this time, extensive vitritis with opacities, pale optic disc, periphlebitis, serous detachment of the macula, retinal hemorrhages, and a whitish subretinal peripheral mass were seen. Following a vitrectomy and irrigation with voriconazole, vision was restored [11].

To date, a few over 20 cases of endogenous endopthalmitis from *S*. *apiospermum* have been reported in the literature (reviewed in Larocco et al. and Table 1). The collection of cases, with a large proportion of systemically ill patients having a wide range of presentations, emphasizes the need to have adequate biopsy results. They also point out the importance of working collaboratively with an infectious disease and internal medicine team. Various antifungal agents have been administered intravitreously for treating fungal endophthalmitis. The most common agent, amphotericin B, is associated with retinal toxicity and resistance has emerged [15, 18]. Amphotericin B is generally ineffective against *S*. *apiospermum* and voriconazole, the second-generation derivative of fluconazole, is accepted as the treatment of choice for this pathogen. It is a broad-spectrum antifungal agent with high bioavailability, quick onset of action, and good ocular penetration [15].

The investigation of the pharmacokinetics of voriconazole indicates that the MIC_90_ (minimum inhibitory concentrations at which 90% of isolates of *S*. *apiospermum* are inhibited) can be attained in the vitreous and aqueous after oral administration [15, 19]. Oral dosing is 200 mg BID with or without a loading dose [15]. Alternatively, it can be given twice a day IV with a loading dose of 6 mg/kg Q12 hours for 1 day, followed by 4 mg/kg BID. While animals studies report a intravitreal voriconazole dose of 100 μg to be effective and safe [20], 200 μg intravitreal injection has been used successfully in humans [15].

## Conclusion

In summary, this is rare a case of endogenous fungal endophthalmitis due to *S*. *apiospermum* in which a history of prior infection in the other eye allowed appropriate early intervention, both pharmacologic and surgical, leading to a successful outcome. The patient was treated aggressively with intravitreal voriconazole, systemic voriconazole, and pars plana vitrectomy leading to a favorable outcome. Thus, it is reasonable to initiate early treatment with voriconazole when fungal endopthalmitis is suspected even prior to obtaining definitive culture results.

## References

[1] Cortez KJ, Roilides E, Quiroz-Telles F, Meletiadis J, Antachopoulos C, Knudsen T, Buchanan W, Milanovich J, Sutton DA, Fothergill A, Rinaldi MG, Shea YR, Zaoutis T, Kottilil S, Walsh TJ (2008). Infections caused by *Scedosporium* spp. Clin Microbiol Rev.

[2] McGuire TW, Bullock JD, Bullock JD, Elder BL, Funkhouser JW (1991). Fungal endophthalmitis. An experimental study with a review of 17 human ocular cases. Arch Ophthalmol.

[3] Rippon JW (1981). Petriellidiosis: the great imitator. Clin Microbiol Newletter.

[4] Musk M, Chambers D, Chin W, Murray R, Gabbay E (2006). Successful treatment of disseminated scedosporium infection in 2 lung transplant recipients: review of the literature and recommendations for management. J Heart Lung Transplant.

[5] Shankar S, Biswas J, Gopal L, Bagyalakshmi R, Therese L, Borse NJ (2007). Anterior chamber exudative mass due to *Scedosporium apiospermum* in an immunocompetent individual. Indian J Ophthalmol.

[6] Nochez Y, Arsene S, Le Guellec C, Bastides F, Morange V, Chaumais MC, Pisella PJ (2008). Unusual pharmacokinetics of intravitreal and systemic voriconazole in a patient with *Scedosporium apiospermum* endophthalmitis. J Ocul Pharmacol Ther.

[7] Chen FK, Chen SD, Tay-Kearney ML (2007). Intravitreal voriconazole for the treatment of endogenous endophthalmitis caused by *Scedosporium apiospermum*. Clin Experiment Ophthalmol.

[8] Sarvat B, Sarria JC (2007). Implantable cardioverter-defibrillator infection due to *Scedosporium apiospermum*. J Infect.

[9] McKelvie PA, Wong EY, Chow LP, Hall AJ (2001). Scedosporium endophthalmitis: two fatal disseminated cases of *Scedosporium* infection presenting with endophthalmitis. Clin Experiment Ophthalmol.

[10] Orr PH, Safneck JR, Napier LB (1993). *Monosporium apiospermum* endophthalmitis in a patient without risk factors for infection. Can J Ophthalmol.

[11] Ikewaki J, Imaizumi M, Nakamuro T, Motomura Y, Ohkusu K, Shinoda K, Nakatsuka K (2009). Peribulbar fungal abscess and endophthalmitis following posterior subtenon injection of triamcinolone acetonide. Acta Ophthalmol.

[12] Larocco A, Barron JB (2005). Endogenous *Scedosporium apiospermum* endophthalmitis. Retina.

[13] Figueroa MS, Fortun J, Clement A, De Arevalo BF (2004). Endogenous endophthalmitis caused by *Scedosporium apiospermum* treated with voriconazole. Retina.

[14] Glassman MI, Henkind P, Alture-Werber E (1973). *Monosporium apiospermum* endophthalmitis. Am J Ophthalmol.

[15] Zarkovic A, Guest S (2007). *Scedosporium apiospermum* traumatic endophthalmitis successfully treated with voriconazole. Int Ophthalmol.

[16] Nulens E, Eggink C, Rijs AJ, Wesseling P, Verweij PE (2003). Keratitis caused by *Scedosporium apiospermum* successfully treated with a cornea transplant and voriconazole. J Clin Microbiol.

[17] Jain A, Egbert P, McCulley TJ, Blumenkranz MS, Moshfeghi DM (2007). Endogenous *Scedosporium apiospermum* endophthalmitis. Arch Ophthalmol.

[18] Axelrod AJ, Peyman GA (1973). Intravitreal amphotericin B treatment of experimental fungal endophthalmitis. Am J Ophthalmol.

[19] Hariprasad SM, Mieler WF, Holz ER, Gao H, Kim JE, Chi J, Prince RA (2004). Determination of vitreous, aqueous, and plasma concentration of orally administered voriconazole in humans. Arch Ophthalmol.

[20] Gao H, Pennesi ME, Shah K, Qiao X, Hariprasad SM, Mieler WF, Wu SM, Holz ER (2004). Intravitreal voriconazole: an electroretinographic and histopathologic study. Arch Ophthalmol.

